# Hypersensitive C-reactive protein-atherogenic index as a novel marker for metabolic dysfunction-associated steatotic liver disease in type 2 diabetes mellitus

**DOI:** 10.3389/fendo.2025.1721278

**Published:** 2025-11-19

**Authors:** Wei Wang, Yan Ying Chen, Xiu Li Guo, Yan Tong

**Affiliations:** National Metabolic Management Center, Longyan First Affiliated Hospital of Fujian Medical University, Longyan, Fujian, China

**Keywords:** hypersensitive C-reactive protein-atherogenic index, metabolic dysfunction-associated steatotic liver disease, C-reactive protein-triglyceride glucose index, triglyceride-glucose index, atherogenic index of plasma, type 2 diabetes mellitus

## Abstract

**Background:**

Hypersensitive C-reactive protein-atherogenic index (CAI) may serve as a novel marker for metabolic dysfunction-associated steatotic liver disease (MASLD) in type 2 diabetes mellitus (T2DM). This study aimed to evaluate the association between CAI and MASLD risk, while comparing its diagnostic performance with C-reactive protein-triglyceride glucose index (CTI), triglyceride-glucose (TyG) index, and atherogenic index of plasma (AIP).

**Method:**

The final cohort included 1,071 individuals with T2DM from the Metabolic Management Center of our hospital. The association between CAI and MASLD was assessed by the binomial logistic regression, restricted cubic splines (RCS), and subgroup analysis. Receiver operating characteristic curve analysis was performed to evaluate the diagnostic performance of CAI for MASLD, with DeLong analysis used to compare its diagnostic ability to CTI, TyG index, and AIP.

**Results:**

Individuals in the higher CAI quartiles demonstrated a greater prevalence of MASLD (*P* < 0.05). After adjusting for confounding factors, CAI was independently associated with a higher risk of MASLD (*OR:* 2.14, 95% CI: 1.74-2.62, *P* < 0.001). Each SD increase in CAI was associated with a 99% higher risk of MASLD (*OR:* 1.99, 95% CI: 1.65-2.39, *P* < 0.001). These associations persisted across subgroups of sex, anti-hepatic steatosis hypoglycemic agent, hypertension, alcohol consumption, and statin use (all *P* < 0.05). RCS analysis revealed a linear association between CAI and risk of MASLD (*P* for nonlinearity = 0.357). ROC analysis indicated that CAI had a diagnostic ability for MASLD (AUC:0.732, 95%CI:0.702-0.762), outperforming CTI (AUC difference: 0.020, 95% CI: 0.007-0.034, *P* = 0.003), TyG (AUC difference: 0.044, 95% CI: 0.026-0.062, *P* < 0.001), and AIP (AUC difference: 0.022, 95% CI: 0.011-0.033, *P* < 0.001) in the DeLong analysis.

**Conclusion:**

The CAI could serve as a novel marker for screening high-risk populations for MASLD in T2DM.

## Introduction

Metabolic dysfunction-associated steatotic liver disease (MASLD), previously named non-alcoholic fatty liver disease (NAFLD) or metabolic dysfunction-associated fatty liver disease (MAFLD), was characterized as excessive triglyceride (TG) accumulation in hepatocytes and has been demonstrated as the leading cause of liver-related morbidity and mortality (1). Current practice guidelines for the management of MASLD emphasize the importance of early diagnosis and preventive strategies in managing MASLD (2, 3). The pathogenesis of MASLD is complex, involving key mechanisms such as insulin resistance and inflammation (4, 5). As a metabolic disorder, MASLD is frequently associated with abnormal metabolic markers, including elevated blood glucose and dyslipidemia. Numerous clinical studies have identified biomarkers related to inflammation and insulin resistance based on these clinical features of MASLD, which help identify high-risk populations. One such biomarker was the triglyceride-glucose (TyG) index, a surrogate marker for insulin resistance first proposed in 2008 by Simental-Mendía et al., which integrates fasting blood glucose (FBG) and triglyceride (TG) levels (6). It has been widely demonstrated to have significant associations with various cardiometabolic disorders like cardiovascular disease (CVD) (7), type 2 diabetes mellitus (T2DM) (8), metabolic syndrome (9), and MASLD (10). Inflammation plays a pivotal role in the development of MASLD, as well as in the broader spectrum of cardiometabolic diseases and certain cancers. Hypersensitive C-reactive protein (hsCRP), a well-established marker of systemic inflammation, has proven to be a reliable indicator of inflammatory activity in these conditions (11, 12). In 2022, Ruan et al. developed a novel marker reflecting systemic inflammation and insulin resistance, known as the C-reactive protein-triglyceride glucose index (CTI) (13). Since its introduction, CTI has been investigated for its associations with cardiometabolic disorders (14, 15) and has proven to be a more effective marker for NAFLD compared to the TyG index (16). However, a concern arises in T2DM, particularly those with fluctuating FBG levels. In these individuals, episodes of extremely high or low blood glucose can reduce the diagnostic reliability of both the TyG index and CTI for identifying high-risk MASLD populations.

The atherogenic index of plasma (AIP) integrates TG and high-density lipoprotein cholesterol (HDL-c), which demonstrated a significant association with CVD (17–19) and MASLD (20). Additionally, Tao et al. found that AIP had the strongest ability to predict the risk of MACCEs in T2DM (21). Compared to FBG, HDL-c is more stable in T2DM patients and is less likely to exhibit extreme fluctuations, making AIP a potentially more effective marker than the TyG index for assessing the risk of cardiometabolic diseases in these individuals. Epidemiological investigation suggests that more than half of T2DM have MASLD (22). In addition to liver damage, MASLD can significantly increase the risk of extrahepatic complications such as CVD and diabetic-related complications (23). The coexistence of MASLD in T2DM patients can accelerate the progression of these comorbidities, highlighting the importance of early detection in T2DM. To better assess MASLD in T2DM, this study developed a novel marker derived from the CTI calculation formula, combining hsCRP and AIP, termed the “Hypersensitive C-reactive protein-atherogenic Index” (CAI). The objective of this study is to evaluate the association between CAI and MASLD risk, while comparing its diagnostic performance with other markers, such as CTI, TyG index, and AIP.

## Study cohort and methods

### Data source

**Figure 1:**
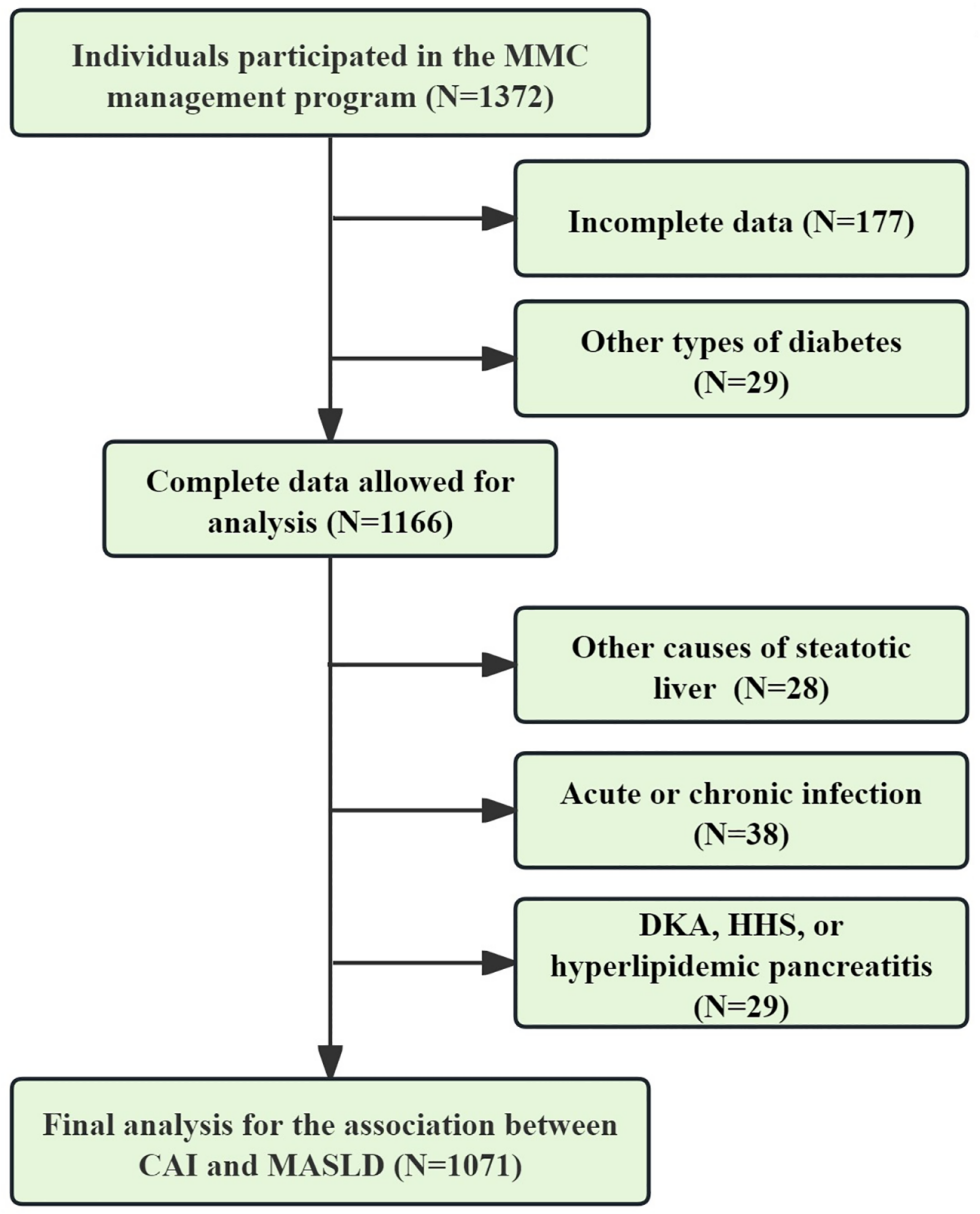
Data selection process for final analysis. CAI, hypersensitive C-reactive protein-atherogenic index; MASLD, metabolic dysfunction-associated steatotic liver disease; DKA, diabetic ketoacidosis; HHS, hyperglycemic hyperosmolar syndrome.

The data utilized for the final analysis were derived from the National Metabolic Management Center (MMC) program, which received approval from the institutional ethics committee in full compliance with the principles outlined in the Declaration of Helsinki (IC-2022-009). All participants were thoroughly informed of the study’s objectives and provided written informed consent. The inclusion criteria for the study were as follows: 1) Diagnosis of T2DM and participation in the MMC program from March 2022 to December 2024; 2) Age ≥ 18 years; 3) Completed data. The exclusion criteria for the study were as follows: 1) Presence of liver comorbidities (e.g., autoimmune hepatitis, viral hepatitis, and liver neoplasms), use of medications (e.g., methotrexate, antipsychotics, estrogens, tamoxifen, antiretroviral drugs, or glucocorticoids), or heavy drinking (daily intake ≥30 g for men and ≥20 g for women), all of which may induce hepatic steatosis; 2) Presence of acute or chronic infections (hsCRP>9 mg/L), or systemic diseases (e.g., hematologic disorders, rheumatic diseases, or malignancies) that could lead to an increase in hsCRP; 3) Presence of severe hyperglycemia or hypertriglyceridemia (e.g., diabetic ketoacidosis, hyperglycemic hyperosmolar syndrome, and hyperlipidemic pancreatitis) that may cause substantial fluctuations in FBG, TG, and HDL-c. Following the application of these inclusion and exclusion criteria, 1,071 participants were eligible for the final analysis (**Figure 1**).

### Study covariates

Demographic information, including age, diabetic duration, sex, alcohol consumption, hypertension, receiving stain, and anti-hepatic steatosis hypoglycemic agent (e.g., thiazolidinediones, sodium glucose cotransporter 2 inhibitors, and glucagon-like peptide-1 receptor agonists) was collected through a structured questionnaire. Anthropometric measurements, waist circumference (WC), systolic blood pressure (SBP), diastolic blood pressure (DBP), and body mass index (BMI), was measured by trained researchers. Following an overnight fast, venous blood samples were carefully obtained by the trained nurses to measure glycated hemoglobin (HbA1c), FBG, hsCRP, uric acid (UA), creatinine, TG, total cholesterol (TC), HDL-c, low-density lipoprotein cholesterol (LDL-c), alanine aminotransferase (ALT), aspartate aminotransferase (AST), alkaline phosphatase, and albumin. Biochemical indices and hsCRP were determined using automated analyzers (Roche Diagnostics Corporation) from fasting venous blood samples. HbA1c levels were measured by high-performance liquid chromatography using a D10 analyzer (Bio-Rad).

### Exposure variable

The exposure variable, CAI, was calculated using the following formula: CAI = 0.412×Ln (hsCRP [mg/L]) + Ln (TG [mg/dL]/HDL-c [mg/dL]). To compare the diagnostic ability between CAI and other biomarkers for MASLD, the following variables were also calculated: CTI = 0.412×Ln (hsCRP [mg/L]) + Ln (TG [mg/dL] × FBG [mg/dL])/2) (13); AIP = log_10_ (TG [mg/dL]/HDL-c [mg/dL]) (24); TyG index=Ln (TG [mg/dL] × FBG [mg/dL])/2) (6).

### Outcome variable

The outcome variable, MASLD, was diagnosed based on the diagnostic criteria recommended by the latest EASL-EASD-EASO guidelines: Diagnosis of MASLD was confirmed in individuals with T2DM who exhibited hepatic steatosis, either detected by imaging or liver biopsy, and absence of other identifiable causes of hepatic steatosis (2). A non-enhanced abdominal CT scan was used to assess hepatic steatosis. The CT scans were reviewed by an experienced radiologist who was blinded to the clinical data and calculated the CT hepato-spleen attenuation measurement (CT_L/S_). CT_L/S_ was calculated using the following formula: mean liver attenuation divided by the mean spleen attenuation. Liver attenuation was measured by averaging the HU values from three 3 cm² circular regions of interest (ROIs) located in the left hepatic lobe, the anterior segment of the right hepatic lobe, and the posterior part of the right hepatic lobe. Splenic attenuation was calculated as the average HU of three 2 cm² ROIs drawn from the upper, middle, and lower thirds of the spleen. Participants with a mean CT_L/S_ value less than 1.0 were categorized as having hepatic steatosis (25).

### Statistical analysis

Statistical analyses were performed using SPSS version 30.0 (IBM, Armonk, NY, USA) and R version 4.2.3. Baseline characteristics across CAI quartiles were compared using one-way analysis of variance for continuous variables and chi-squared (χ²) tests for categorical variables. To assess the associations between CAI and CT_L/S_, as well as the risk of MASLD, multiple linear regression, binomial logistic regression, and restricted cubic splines (RCS) analyses were conducted. Subgroup analyses were performed to investigate potential effect modifications by sex (men vs. women), anti-hepatic steatosis agents (with vs. without), hypertension (with vs. without), alcohol consumption (with vs. without), and statin use (with vs. without). These analyses were adjusted for relevant confounders identified in previous studies (26, 27) across three models to minimize bias: 1) Model 1 (unadjusted); 2) Model 2 (adjusted for age, gender, diabetes duration, alcohol consumption, statin use, and anti-hepatic steatosis hypoglycemic agents); and 3) Model 3 (further adjusted for metabolic parameters, including SBP, DBP, WC, BMI, UA, TC, LDL-c, HbA1c, and creatinine). Receiver operating characteristic curve analysis was performed to evaluate the diagnostic accuracy of CAI for MASLD. Additionally, diagnostic performance comparisons between CAI, CTI, the TyG index, and AIP were conducted using the DeLong test. To address the possibility that CT may miss mild cases of hepatic steatosis, a sensitivity analysis was performed by lowering the diagnostic threshold of CT_L/S_ to 1.1, thereby enhancing the robustness of the findings. A two-tailed *P-value* < 0.05 was considered statistically significant.

## Results

### Baseline characteristics of the study cohort

**Figure 2:**
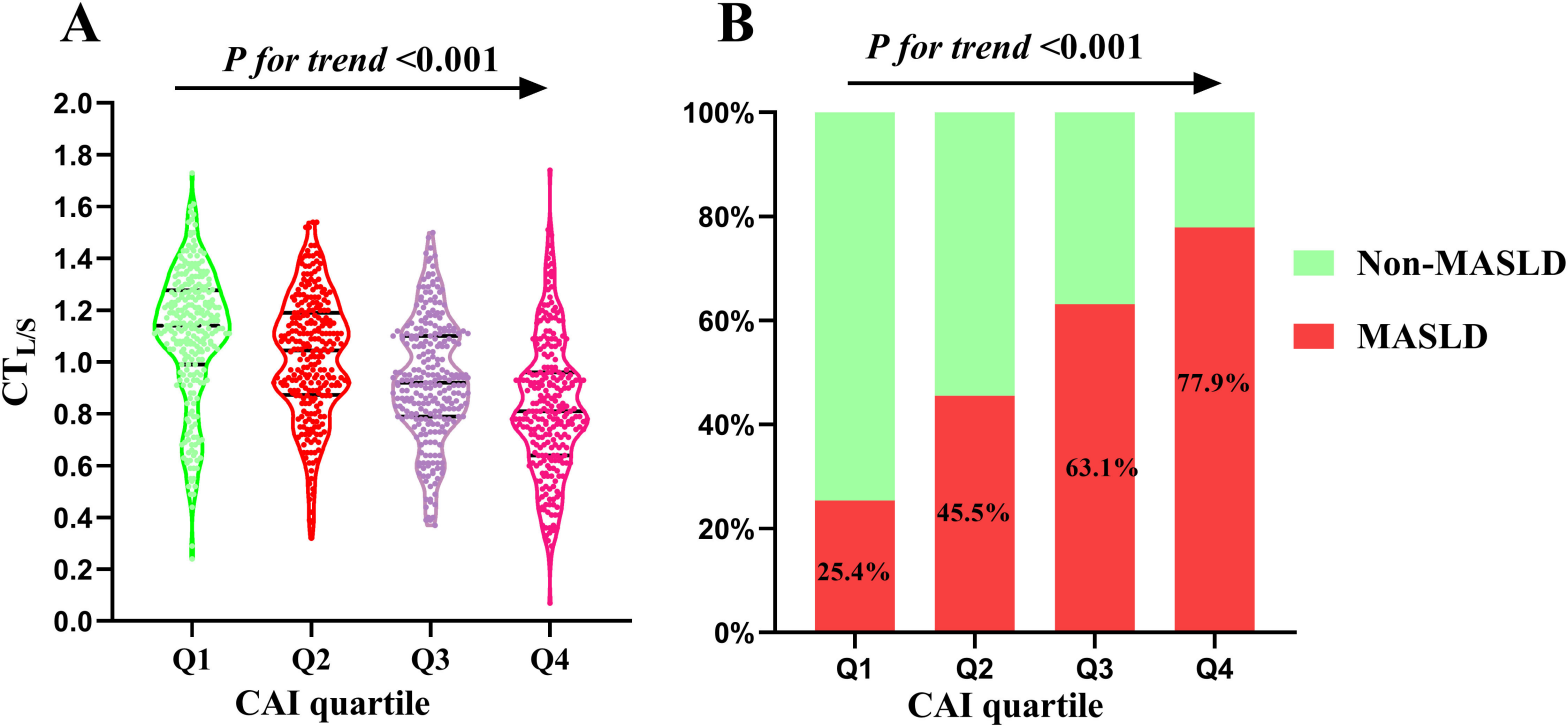
Distribution of CT_L/S_**(A)** and prevalence of MASLD **(B)** across the CAI quartiles. CAI, hypersensitive C-reactive protein-atherogenic index; MASLD, metabolic dysfunction-associated steatotic liver disease; CT_L/S_, CT hepato-spleen attenuation measurement.

The study cohort comprised 1,071 participants, with 56.2% being men, a mean age of 53.5 ± 9.0 years, and 567 (52.9%, 95%CI:49.9%-55.9%) participants diagnosed with MASLD. **Table 1** outlines the baseline characteristics of the study cohort stratified by CAI quartile. Notable differences were observed across quartiles in terms of BMI, WC, SBP, DBP, FBG, HsCRP, TG, TC, LDL-c, HDL-c, UA, creatinine, ALT, AST, ALP, and prevalence of hypertension, all showing statistically significant differences (all *P* < 0.05). Individuals in the higher CAI quartiles exhibited lower levels of CTL/S (**Figure 2A**). Additionally, the prevalence of MASLD increased with higher CAI quartiles. Specifically, the prevalence of MASLD was 25.5% (95%CI: 20.5%-30.9%) in Q1, 45.5% (95%CI: 39.7%-51.5%) in Q2, 63.1% (95%CI: 58.7%-68.6%) in Q3, and 77.9% (95%CI: 72.5%-82.5%) in Q4 (**Figure 2B**).

**Table 1:** Baseline characteristics of the study cohort stratified by CAI quartile.

Characteristics	CAI quartile				*P* value
	Q1 (<1.18)	Q2 (1.19-1.82)	Q3 (1.83-2.41)	Q4 (>2.41)	
Age (year)	53.6 ± 8.9	53.8 ± 8.9	53.3 ± 9.2	53.7 ± 9.0	0.905
Diabetic duration (year)	5.4 ± 3.5	5.2 ± 2.9	5.1 ± 3.2	5.4 ± 3.0	0.475
BMI (kg/m^2^)	22.4 ± 3.1	23.8 ± 2.4	24.9 ± 2.6	26.7 ± 3.6	<0.001
WC (cm)	82.3 ± 8.1	84.8 ± 6.2	87.6 ± 7.4	92.1 ± 8.6	<0.001
SBP (mmHg)	120.9 ± 15.0	126.9 ± 16.4	133.4 ± 14.3	142.9 ± 19.6	<0.001
DBP (mmHg)	78.2 ± 9.9	79.3 ± 9.0	82.5 ± 10.9	86.8 ± 9.7	<0.001
HbA1c (%)	9.0 ± 1.9	9.1 ± 1.6	9.1 ± 1.7	9.2 ± 1.5	0.773
FBG (mg/dL)	146.7 ± 53.7	161.8 ± 53.1	175.5 ± 75.5	179.4 ± 59.3	<0.001
HsCRP (mg/L)	2.06 ± 1.44	3.30 ± 1.42	3.73 ± 1.27	4.73 ± 3.15	<0.001
TG (mg/dL)	84.9 ± 30.9	134.0 ± 40.2	198.6 ± 50.8	348.2 ± 136.1	<0.001
TC (mg/dL)	191.9 ± 45.6	201.4 ± 44.0	205.9 ± 47.2	213.2 ± 50.6	<0.001
HDL-c (mg/dL)	51.8 ± 8.4	44.4 ± 7.2	38.6 ± 5.2	33.2 ± 5.0	<0.001
LDL-c (mg/dL)	122.1 ± 35.9	132.6 ± 33.4	136.5 ± 35.5	138.6 ± 37.0	<0.001
UA (umol/L)	287.8 ± 73.0	335.1 ± 74.0	362.4 ± 78.2	391.1 ± 91.6	<0.001
Creatinine (umol/L)	66.3 ± 15.8	68.7 ± 13.9	70.6 ± 17.5	69.8 ± 17.4	0.014
ALT (IU/L)	31.9 ± 19.7	34.4 ± 16.0	35.6 ± 20.5	37.5 ± 15.4	<0.001
AST (IU/L)	26.1 ± 12.6	28.4 ± 10.4	30.1 ± 12.4	31.9 ± 14.1	<0.001
ALT/AST	1.21 ± 0.44	1.22 ± 0.39	1.21 ± 0.38	1.25 ± 0.45	0.738
ALP (U/L)	76.9 ± 22.5	80.8 ± 22.1	82.5 ± 19.9	83.7 ± 22.5	0.002
Albumin (g/L)	40.5 ± 2.8	40.5 ± 2.2	40.5 ± 2.4	40.3 ± 2.1	0.371
Statin, n (%)					
With	81 (30.2)	88 (32.8)	78 (29.1)	90 (33.7)	0.628
Without	187 (69.8)	180 (67.2)	190 (70.9)	177 (67.3)
Sex, n (%)					
Men	152 (56.2)	142 (53.0)	136 (50.7)	172 (64.4)	0.008
Women	116 (43.8)	126 (47.0)	132 (49.3)	95 (35.6)
Alcohol consumption, n (%)					
With	87 (32.5)	92 (34.3)	85 (31.7)	97 (36.3)	0.676
Without	181 (67.5)	176 (65.7)	183 (68.3)	170 (63.7)
Hypertension, n (%)					
With	63 (23.5)	63 (23.5)	99 (36.9)	156 (58.4)	<0.001
Without	205 (76.5)	205 (76.5)	169 (63.1)	111 (41.1)
Anti-hepatic steatosis hypoglycemic agent, n (%)					
With	78 (29.1)	84 (31.3)	92 (34.3)	100 (37.5)	0.189
Without	190 (70.9)	184 (68.7)	176 (65.7)	167 (62.5)

Data are expressed as n (%) or mean ± standard deviation.

CAI, hypersensitive C-reactive protein-Atherogenic index; BMI, body mass index; WC, waist circumference; SBP, systolic blood pressure; DBP, diastolic blood pressure; HbA1c, glycated hemoglobin; FBG, fasting blood glucose; HsCRP, hypersensitive C-reactive protein; TG, triglyceride; TC, total cholesterol; HDL-c, high-density lipoprotein cholesterol; LDL-c, low-density lipoprotein cholesterol; UA, uric acid; ALT, alanine aminotransferase; AST, aspartate aminotransferase; ALP, alkaline phosphatase.

### Associations between CAI, CT_L/S_, and risk of MASLD

**Figure 3:**
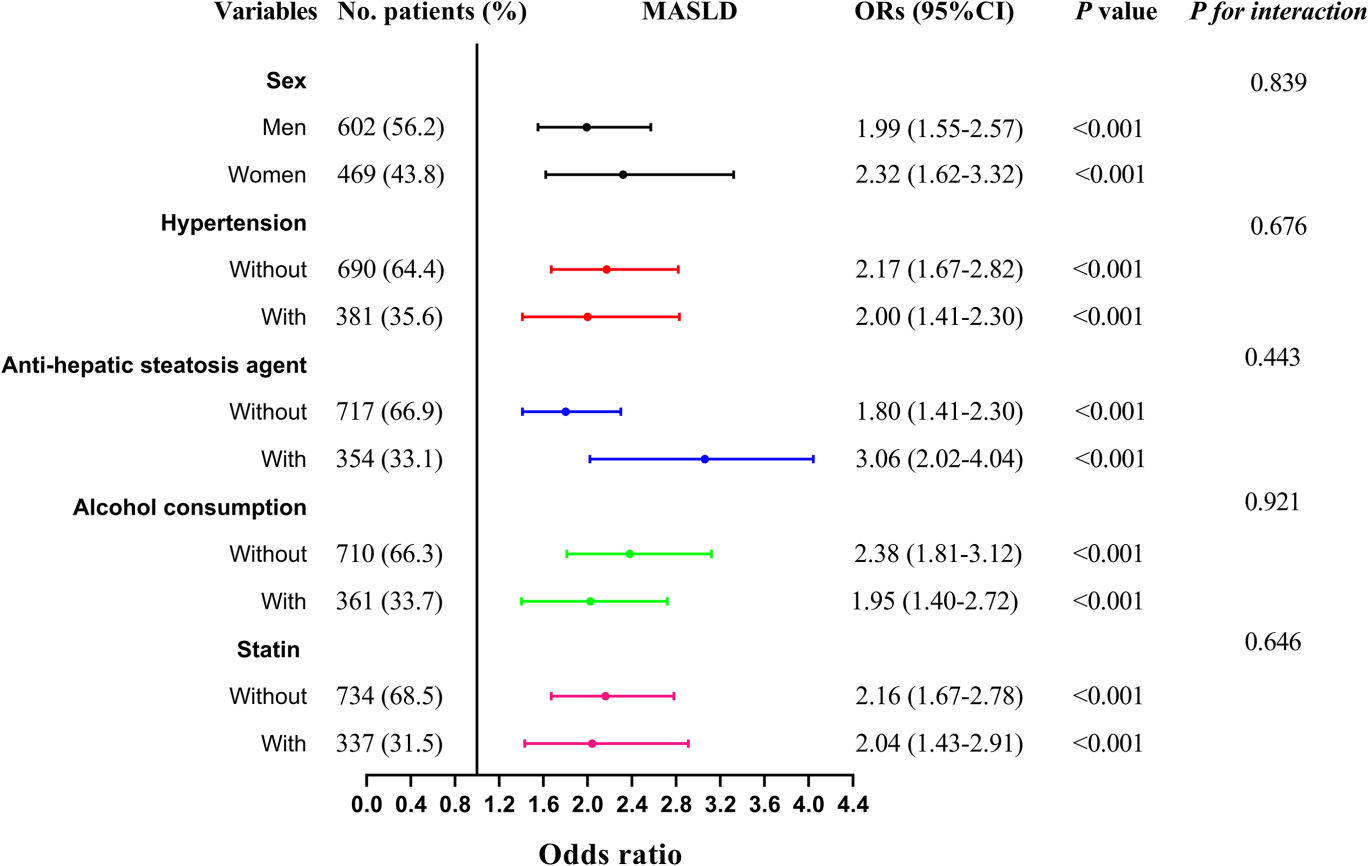
Subgroup analysis stratified by sex (men vs. women), anti-hepatic steatosis agents (with vs. without), hypertension (with vs. without), alcohol consumption (with vs. without), and statin use (with vs. without) for the association between CAI and MASLD. Model adjusted for age, gender, diabetic duration, alcohol consumption, statin, receiving anti-hepatic steatosis hypoglycemic agent, and metabolic profiles like systolic blood pressure, diastolic blood pressure, waist circumference, body mass index, uric acid, total cholesterol, low-density lipoprotein cholesterol, glycated hemoglobin, and creatinine. CAI, hypersensitive C-reactive protein-atherogenic index; MASLD, metabolic dysfunction-associated steatotic liver disease.

**Figure 4:**
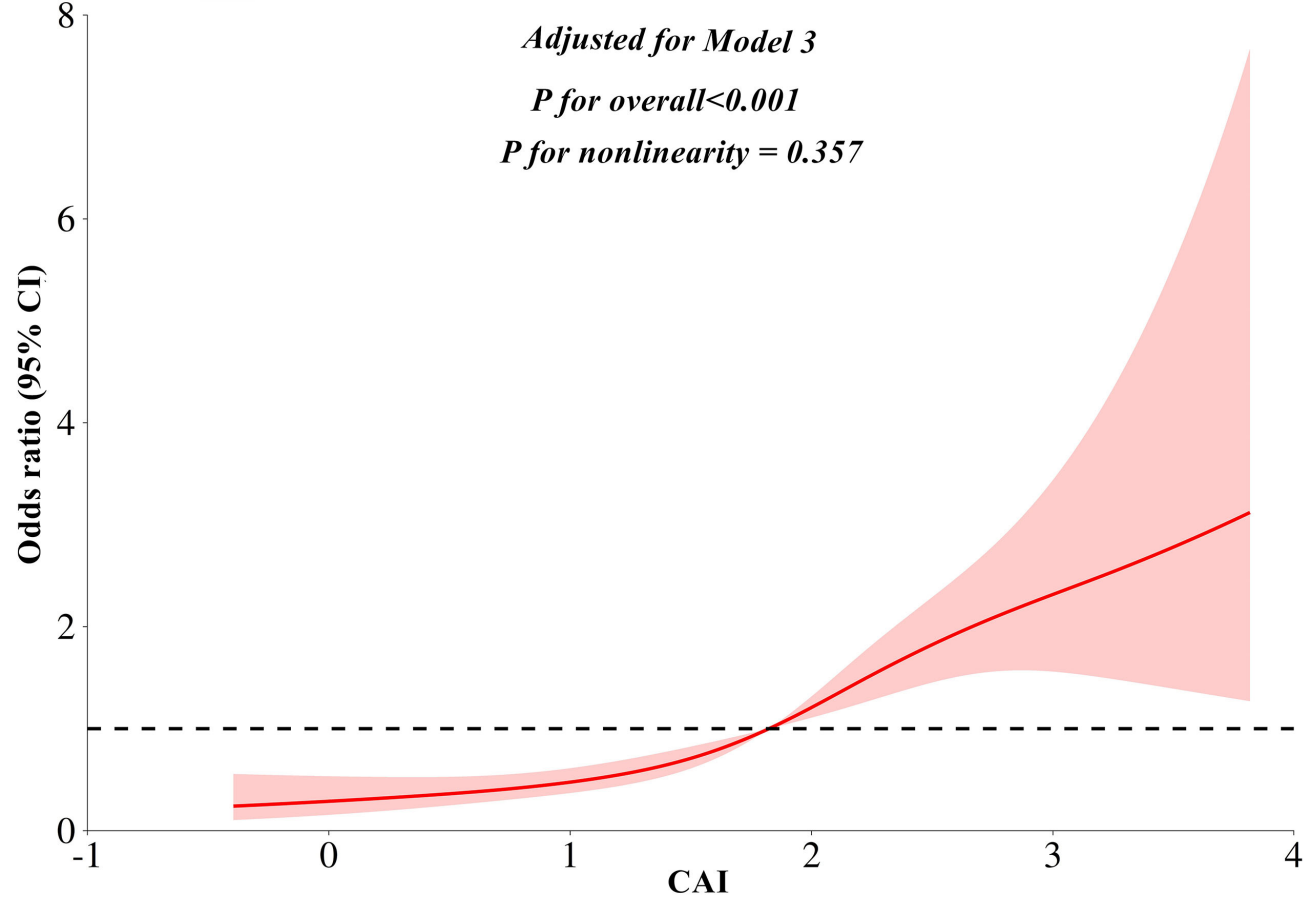
Restricted cubic splines analyses for the association between CAI and MASLD. Model adjusted for age, gender, diabetic duration, alcohol consumption, statin, receiving anti-hepatic steatosis hypoglycemic agent, and metabolic profiles like systolic blood pressure, diastolic blood pressure, waist circumference, body mass index, uric acid, total cholesterol, low-density lipoprotein cholesterol, glycated hemoglobin, and creatinine. CAI, hypersensitive C-reactive protein-atherogenic index; MASLD, metabolic dysfunction-associated steatotic liver disease.

The multivariate linear regression analysis revealed a significant negative association between the CAI and CT_L/S_ in both Model 1 (*β* = -0.399, *P* < 0.001) and Model 2 (*β* = -0.395, *P* < 0.001). This association persisted even after full adjustments in Model 3 (*β* = -0.286, *P* < 0.001). As shown in **Table 2**, In the binomial logistic regression analysis examining the relationship between CAI and the risk of MASLD, participants in the higher quartiles of CAI had an increased risk of MASLD compared to those in the first quartile across all models (*P* < 0.001). After full adjustments in Model 3, CAI was independently associated with a higher risk of MASLD (*OR:* 2.14, 95% CI: 1.74-2.62, *P* < 0.001). Each SD increase in CAI was associated with a 99% higher risk of MASLD (*OR:* 1.99, 95% CI: 1.65-2.39, *P* < 0.001). As shown in **Figure 3**, Subgroup analysis indicated that the independent association between CAI and the risk of MASLD persisted in subgroups stratified by sex, anti-hepatic steatosis hypoglycemic agent, hypertension, alcohol consumption, and statin (all *P* < 0.05). Moreover, RCS analysis also exhibited a linear association between CAI and risk of MASLD (*P* for nonlinearity = 0.357) (**Figure 4**).

**Table 2:** Binomial logistic regression analysis for the association between CAI and the prevalence of MASLD.

Variable	Model 1		Model 2		Model 3	
	OR (95%CI)	*P* value	OR (95%CI)	*P* value	OR (95%CI)	*P* value
Overall	2.74(2.32-3.24)	<0.001	2.77(2.34-3.27)	<0.001	2.14(1.74-2.62)	<0.001
Per SD increase	2.50(2.15-2.90)	<0.001	2.51(2.16-2.93)	<0.001	1.99(1.65-2.39)	<0.001
Q1	Ref. (1.0)		Ref. (1.0)		Ref. (1.0)	
Q2	2.40(1.66-3.46)	<0.001	2.44(1.69-3.53)	<0.001	2.05(1.37-3.07)	<0.001
Q3	4.98(3.44-7.20)	<0.001	5.12(3.52-7.43)	<0.001	3.38(2.20-5.19)	<0.001
Q4	10.07(6.75-15.02)	<0.001	10.22(6.83-15.28)	<0.001	5.22(3.19-8.54)	<0.001
*P* for trend	<0.001		<0.001		<0.001	

Model 1 was unadjusted. Model 2 adjusted for age, gender, diabetic duration, alcohol consumption, statin, and receiving anti-hepatic steatosis hypoglycemic agent. Model 3 adjusted for age, gender, diabetic duration, alcohol consumption, statin, receiving anti-hepatic steatosis hypoglycemic agent, and metabolic profiles like systolic blood pressure, diastolic blood pressure, waist circumference, body mass index, uric acid, total cholesterol, low-density lipoprotein cholesterol, glycated hemoglobin, and creatinine.

CAI, C-reactive protein-Atherogenic index; MASLD, metabolic dysfunction-associated steatotic liver disease.

### Comparison of diagnostic ability between CAI, CTI, TyG, and AIP

**Figure 5:**
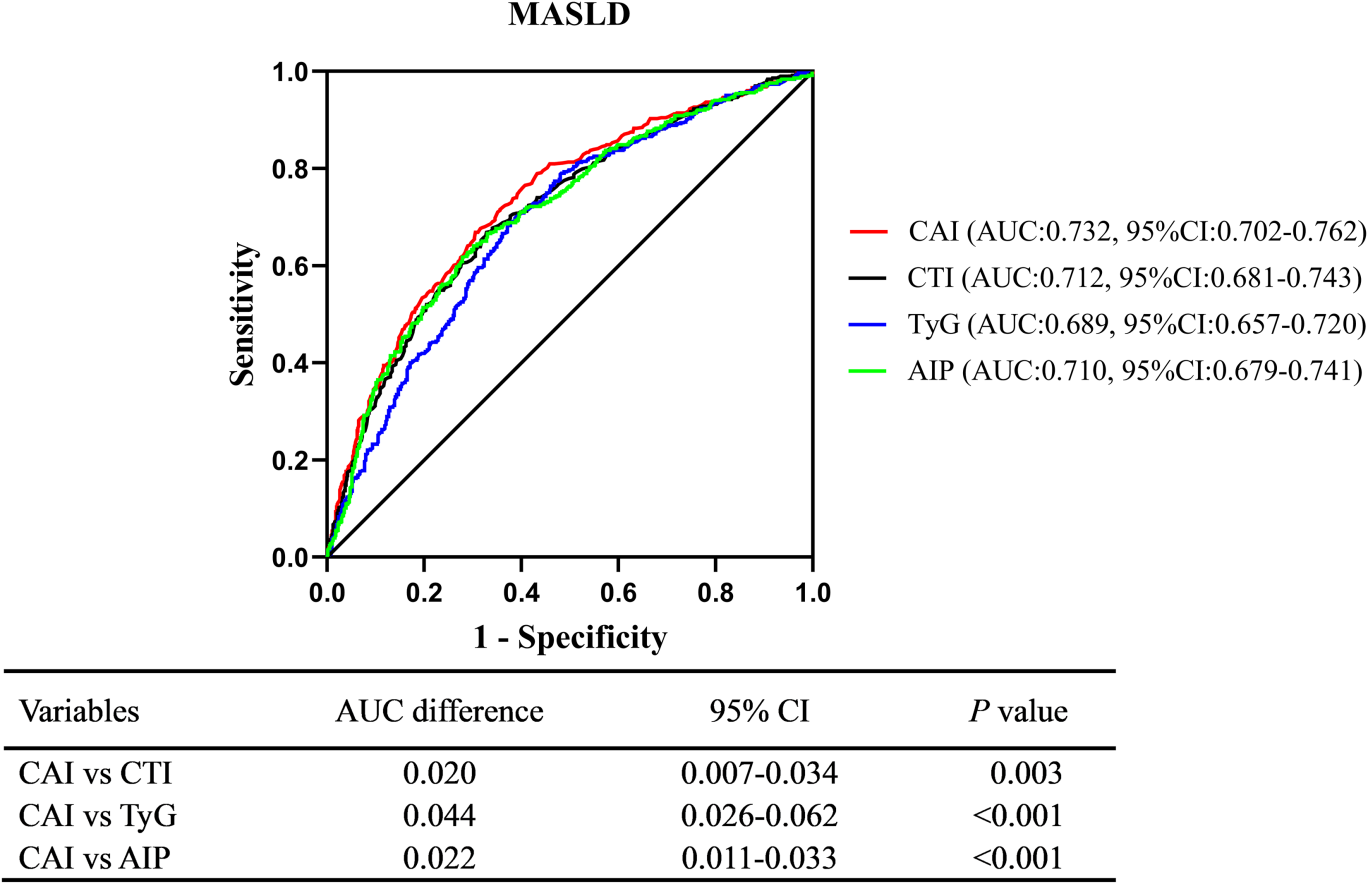
Comparison of diagnostic ability between CAI, CTI, TyG, and AIP using the DeLong analysis. CAI, hypersensitive C-reactive protein-atherogenic index; CTI, C-reactive protein-triglyceride glucose index; TyG, triglyceride-glucose index; AIP, atherogenic index of plasma; MASLD, metabolic dysfunction-associated steatotic liver disease.

ROC analysis indicated that CAI had a moderate diagnostic ability for MASLD, with an AUC (95% CI) of 0.732 (0.702-0.762) and an optimal cut-off value of 1.77, corresponding to a sensitivity of 69.5% and a specificity of 66.9%. Moreover, CAI had better diagnostic performance than CTI (AUC difference: 0.020, 95% CI: 0.007-0.034, *P* = 0.003), TyG index (AUC difference: 0.044, 95% CI: 0.026-0.062, *P* < 0.001), and AIP (AUC difference: 0.022, 95% CI: 0.011-0.033, *P* < 0.001) in the DeLong analysis (**Figure 5**).

### Sensitivity analysis

Given that CT may fail to detect mild cases of hepatic steatosis, we conducted a sensitivity analysis to investigate the aforementioned relationship. The results revealed that CAI remained significantly associated with MASLD after adjustment for Model 3 (*OR:* 2.08, 95% CI: 1.70-2.55, *P* < 0.001). Each SD increase in CAI corresponded to a 94% increased risk of MASLD (*OR:* 1.94, 95% CI: 1.62-2.34, *P* < 0.001). As shown in **Supplementary Figure 1**, the linear association between CAI and the risk of MASLD persisted (*P* for nonlinearity = 0.301). Furthermore, CAI also exhibited superior diagnostic performance than CTI (AUC difference: 0.020, 95% CI: 0.005-0.034, *P* = 0.009), TyG index (AUC difference: 0.046, 95% CI: 0.026-0.066, *P* < 0.001), and AIP (AUC difference: 0.026, 95% CI: 0.014-0.038, *P* < 0.001) (**Supplementary Figure 2**).

## Discussion

T2DM and MASLD represent two of the most significant global health challenges. Given the shared pathophysiological mechanisms between T2DM and MASLD, the prevalence of MASLD is notably high in T2DM. Due to the substantial adverse impact of MASLD on T2DM, the development of reliable biomarkers for screening high-risk populations is of paramount importance. CAI, a novel marker that integrates HsCRP and AIP, has emerged as a potential biomarker for MASLD. This study evaluated the association between CAI and MASLD risk and compared its diagnostic performance with other markers. Our findings revealed a negative association between CAI and CT_L/S_ after adjusting for confounding factors. Further analyses, including binomial logistic regression, restricted cubic spline (RCS) analysis, and subgroup analysis, confirmed an independent relationship between CAI and the risk of MASLD. Moreover, CAI demonstrated superior diagnostic accuracy compared to the CTI, TyG index, and AIP, as assessed by DeLong analysis. Sensitivity analysis, in which the diagnostic threshold of CT_L/S_ was lowered to 1.1, yielded consistent results.

T2DM frequently contributes to the onset of insulin resistance and systemic inflammation, both of which are key factors that elevate the risk of developing MASLD. The coexistence of MASLD and T2DM not only escalates the risk of liver-related complications but also amplifies the likelihood of extrahepatic adverse outcomes. Evidence from various cohort studies has consistently shown that hepatic steatosis significantly increases the risk of myocardial infarction, ischemic stroke, heart failure, CVD, chronic kidney disease, and all-cause mortality (28–30). While lifestyle modifications and exercise interventions are critical in managing MASLD, there are currently limited pharmacological options approved for treatment. Moreover, diagnosis of MASLD typically relies on the identification of hepatic steatosis by imaging techniques or liver biopsy, which are often costly or invasive, limiting their widespread application. These challenges have spurred a significant number of studies aimed at identifying non-invasive blood markers for the early detection of high-risk MASLD populations. However, there is a paucity of such markers specifically for T2DM. The TyG index and AIP are key markers of insulin resistance, consistently linked to MASLD, and have emerged as promising biomarkers for identifying individuals at high risk. Several clinical studies have shown that the TyG-related index is significantly associated with MASLD, as well as with all-cause and cardiovascular mortality (31, 32). Furthermore, it has demonstrated good diagnostic performance for MASLD, with AUC values ranging from 0.724 to 0.80 in the general population (10, 33, 34). However, the diagnostic performance of the TyG index has been somewhat diminished in T2DM, with AUC values ranging from 0.651 to 0.755 (35–37). This reduced performance may be attributed to the considerable fluctuations in FBG levels in diabetes, which can lead to extreme hyperglycemia and hypoglycemia, affecting the accuracy of the TyG index.

Cardiometabolic diseases are frequently characterized by elevated TG and reduced HDL-c levels. Consequently, TG/HDL-c and its derived parameter “AIP” have emerged as reliable markers of insulin resistance. These indices have been robustly linked to MASLD, with substantial evidence supporting their significant association in T2DM. For instance, Li et al. found that the TG/HDL-c was independently associated with NAFLD in T2DM, with an AUC of 0.732 (38). Similarly, Lin et al. demonstrated a significant association between AIP and NAFLD in T2DM, reporting an AUC of 0.849 (39). Moreover, our previous study also found that TG/HDL-c outperformed the TyG index in identifying MAFLD in T2DM (AUC: 0.742 vs. 0.694, *P* < 0.001) (40). In alignment with these findings, the present study revealed that AIP exhibited superior diagnostic performance compared to the TyG index in T2DM (AUC: 0.710 vs. 0.689, *P* = 0.008). These results suggest that both the TG/HDL-c and AIP may serve as valuable tools for diagnosing MASLD, offering enhanced diagnostic accuracy over the TyG index in the T2DM population. One potential explanation for this improved diagnostic performance is that HDL-c is more stable and less susceptible to fluctuations in blood glucose levels compared to FBG, particularly in individuals with T2DM. The CTI, which combines HsCRP and the TyG index, has been shown to be significantly associated with MASLD. Zhou et al. demonstrated a notable correlation between the CTI and the prevalence of NAFLD and liver fibrosis. Furthermore, the CTI exhibited superior diagnostic performance compared to the TyG index (AUC:0.756 vs. 0.739) (16). In line with the findings of the TyG index, our study found a diminished diagnostic value of CTI for MASLD in T2DM, with an AUC of 0.712. Consistent with CTI, CAI also exhibited a significant correlation with the risk of MASLD. Using DeLong analysis, we compared the diagnostic capabilities of CAI with those of the three aforementioned indexes in identifying MASLD. The results indicated that CAI demonstrated superior diagnostic performance compared to CTI, TyG, and AIP, with an AUC of 0.732. Since CT may fail to detect mild hepatic steatosis, potentially underestimating the overall incidence of MASLD, this could affect the relationship between CAI and MASLD. In the sensitivity analysis, we lowered the diagnostic threshold of CT_L/S_ to 1.1 to capture mild cases of hepatic steatosis, and the results remained consistent. Given the simplicity of calculating the CAI and the accessibility of hsCRP, TG, and HDL-c markers, CAI may emerge as a cost-effective and efficient tool for clinicians to identify high-risk populations for MASLD in T2DM. Although an independent association between CAI and MASLD has been identified, further investigation is needed to determine whether CAI is linked to the risk of progression to liver fibrosis in MASLD and whether it possesses predictive value. Fibrosis-4 (FIB-4), aspartate aminotransferase to platelet ratio index, and NAFLD fibrosis score were recommended by guidelines as tools for predicting the risk of future liver fibrosis. Recent studies have demonstrated that FIB-4, when combined with indices such as ALBI score and Creatinine-to-Cystatin C ratio, can enhance its predictive capability for liver fibrosis and liver failure (41, 42). Based on these findings, combining CAI with these indices may also improve its predictive ability. However, this hypothesis needs to be confirmed through future cohort studies. Notably, CAI was derived from the CTI calculation formula rather than through a specific model analysis. The optimal predictive model, incorporating hsCRP, TG, and HDL-c, remains to be further explored in future studies.

This study presents several notable strengths, particularly in the development of a novel marker for MASLD in T2DM. However, several limitations should be considered. First, the independent associations between CAI, CT_L/S_, and MASLD were based on a cross-sectional design, which does not capture the long-term effects or causal relationships between CAI and the incidence of MASLD. Future longitudinal studies would be beneficial to confirm these findings. Second, the study population was limited to individuals with T2DM, so further validation in a more diverse cohort is needed to assess the broader clinical applicability of the results. Third, hepatic steatosis was assessed using non-enhanced CT. Future studies incorporating additional detection methods, such as ultrasound CAP, biopsy, or MRI-PDFF, could improve the generalizability of the findings.

## Conclusion

CAI demonstrated an independent association with CT_L/S_ and risk of MASLD. Notably, CAI also exhibited superior diagnostic performance compared to CTI, TyG, and AIP. These findings indicated that CAI may offer a reliable and cost-effective marker for screening high-risk populations for MASLD in T2DM.

## Data Availability

The original contributions presented in the study are included in the article/supplementary material. Further inquiries can be directed to the corresponding author.

## Supplementary material

Supplementary Figure 1 Restricted cubic splines analyses for the association between CAI and MASLD in the sensitivity analysis. Model adjusted for age, gender, diabetic duration, alcohol consumption, statin, receiving anti-hepatic steatosis hypoglycemic agent, and metabolic profiles like systolic blood pressure, diastolic blood pressure, waist circumference, body mass index, uric acid, total cholesterol, low-density lipoprotein cholesterol, glycated hemoglobin, and creatinine. CAI, hypersensitive C-reactive protein-atherogenic index; MASLD, metabolic dysfunction-associated steatotic liver disease.

Supplementary Figure 2 Comparison of diagnostic ability between CAI, CTI, TyG, and AIP using the DeLong analysis in the sensitivity analysis. CAI, hypersensitive C-reactive protein-atherogenic index; CTI, C-reactive protein-triglyceride glucose index; TyG, triglyceride-glucose index; AIP, atherogenic index of plasma; MASLD, metabolic dysfunction-associated steatotic liver disease.
