# Supplementary figures and images for: Hypersensitive C-reactive protein-atherogenic index as a novel marker for metabolic dysfunction-associated steatotic liver disease in type 2 diabetes mellitus
Source: Front Endocrinol (Lausanne). 2025 Nov 19;16:1721278. doi: 10.3389/fendo.2025.1721278 (PMC12672254; doi:10.3389/fendo.2025.1721278)

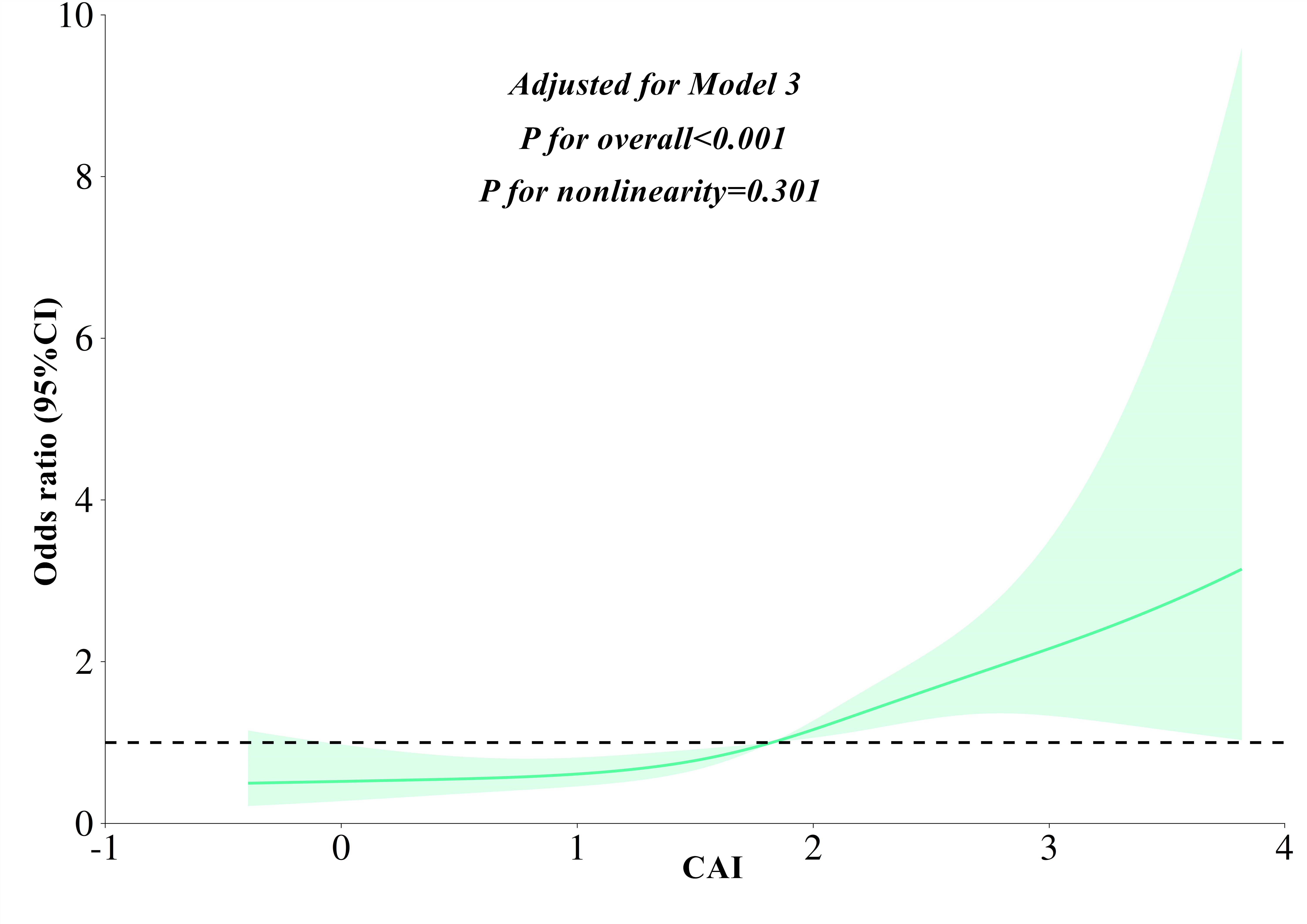

Supplement: Supplementary Figure 1 — Restricted cubic splines analyses for the association between CAI and MASLD in the sensitivity analysis. Model adjusted for age, gender, diabetic duration, alcohol consumption, statin, receiving anti-hepatic steatosis hypoglycemic agent, and metabolic profiles like systolic blood pressure, diastolic blood pressure, waist circumference, body mass index, uric acid, total cholesterol, low-density lipoprotein cholesterol, glycated hemoglobin, and creatinine. CAI, hypersensitive C-reactive protein-atherogenic index; MASLD, metabolic dysfunction-associated steatotic liver disease. [file Image1.tif]

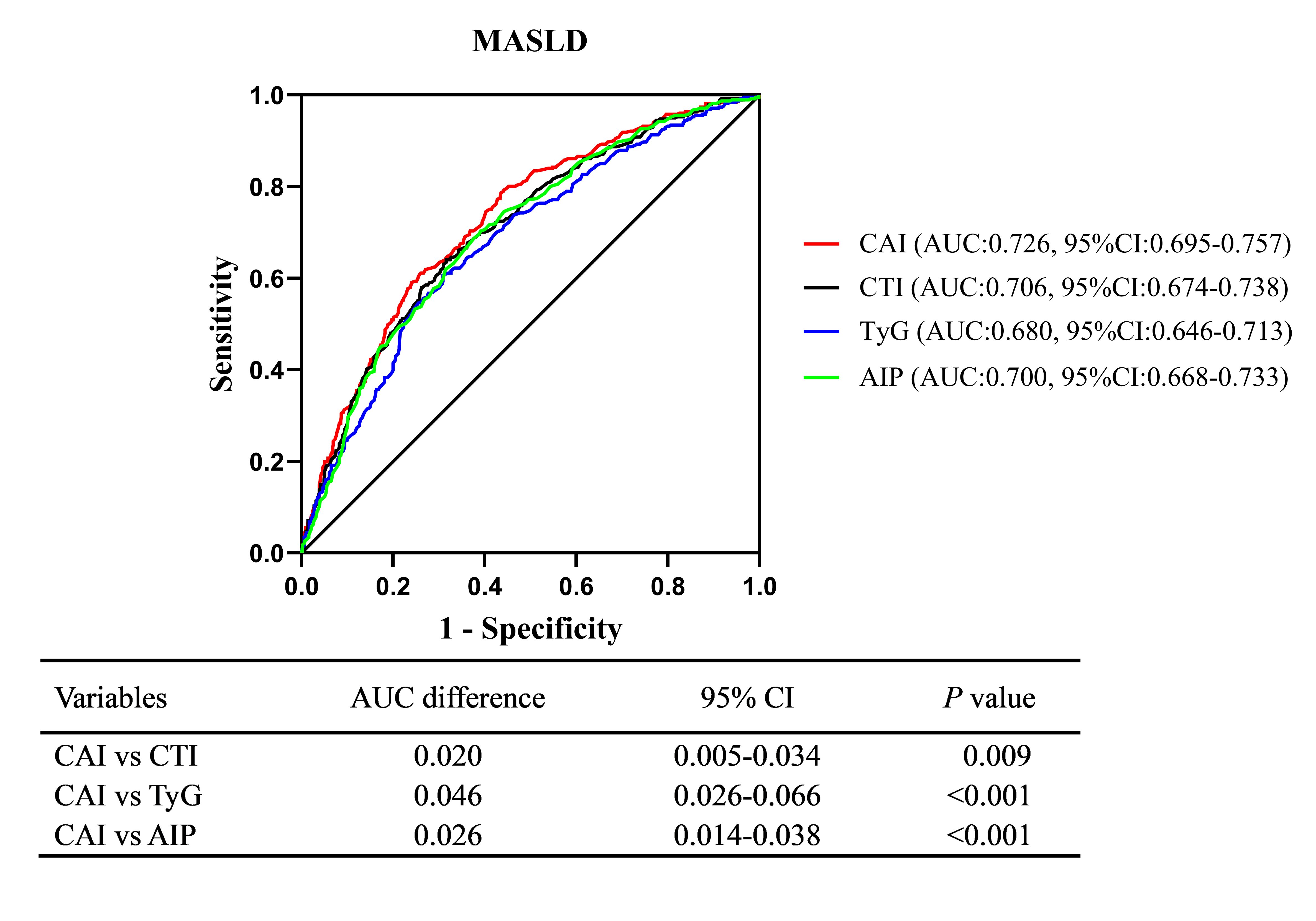

Supplement: Supplementary Figure 2 — Comparison of diagnostic ability between CAI, CTI, TyG, and AIP using the DeLong analysis in the sensitivity analysis. CAI, hypersensitive C-reactive protein-atherogenic index; CTI, C-reactive protein-triglyceride glucose index; TyG, triglyceride-glucose index; AIP, atherogenic index of plasma; MASLD, metabolic dysfunction-associated steatotic liver disease. [file Image2.tif]
